# Effect of a pedometer-based walking intervention on body composition in patients with ESRD: a randomized controlled trial

**DOI:** 10.1186/s12882-020-01753-5

**Published:** 2020-03-16

**Authors:** Anoop Sheshadri, Piyawan Kittiskulnam, Jennifer C. Lai, Kirsten L. Johansen

**Affiliations:** 1 Division of Nephrology, Department of Medicine, University of California, San Francisco, USA; 2 San Francisco Veterans Affairs Medical Center, San Francisco, California USA; 3 Division of Internal Medicine-Nephrology, Department of Medicine, Faculty of Medicine, Chulalongkorn University, Bangkok, Thailand; 4 Special Task Force for Activating Research in Renal Nutrition, (Renal Nutrition Research Group), Office of Research Affairs, Chulalongkorn University, Bangkok, Thailand; 5 Division of Gastroenterology/Hepatology, Department of Medicine, University of California, San Francisco, USA; 6 Division of Nephrology, Hennepin County Medical Center, Minneapolis, MN USA; 7 Division of Nephrology, University of Minnesota, Minneapolis, MN USA

**Keywords:** Physical activity, Body composition, fat mass, muscle mass, Dialysis, Sarcopenia

## Abstract

**Background:**

A randomized trial of a pedometer-based intervention with weekly activity goals led to a modest increase in step count among dialysis patients. In a secondary analysis, we investigated the effect of this intervention on body composition.

**Methods:**

Sixty dialysis patients were randomized to standard care or a 6-month program consisting of 3 months of pedometers and weekly step count targets and 3 months of post-intervention follow-up. We obtained bioelectrical impedance spectroscopy (BIS) data on 54 of these patients (28 control, 26 intervention) and used linear mixed-modeling (adjusted for sex and dialysis modality) to estimate differences in change in total-body muscle mass (TBMM) adjusted for height^2^, fat mass (kg), and body mass index (BMI) (kg/m^2^) between control and intervention groups.

**Results:**

The median age of participants was 57.5 years (53–66), and 76% were men. At baseline, there was no significant difference between groups in age, BMI, race, or body composition, but there were more men in the intervention group. After 3 months, patients in the intervention group increased their average daily steps by 2414 (95% CI 1047, 3782) more than controls (*p* < 0.001), but there were no significant differences in body composition. However, at 6 months, participants in the intervention had a significantly greater increase from baseline in TBMM of 0.7 kg/m^2^ (95% CI 0.3, 1.13), decrease in fat mass (− 4.3 kg [95% CI -7.1, − 1.5]) and decrease in BMI (− 1.0 kg/m^2^ [95% CI -1.8, − 0.2]) relative to controls. In post-hoc analysis, each increase of 1000 steps from 0 to 3 months was associated with a 0.3 kg decrease in fat mass (95% CI 0.05, 0.5) from 0 to 6 months, but there was no dose-response relationship with TBMM/ht^2^ or BMI.

**Conclusion:**

A pedometer-based intervention resulted in greater decreases in fat mass with relative preservation of muscle mass, leading to a greater decrease in BMI over time compared with patients not in the intervention. These differences were driven as much by worsening in the control group as by improvement in the intervention group. Step counts had a dose-response relationship with decrease in fat mass.

**Trial registration:**

ClinicalTrials.gov (NCT02623348). 02 December 2015.

## Background

Patients with end-stage renal disease (ESRD) on dialysis have protein-energy imbalances [1] that can lead to muscle wasting [2]. Lower muscle mass [3, 4] in dialysis patients is associated with impairments in physical function [5]. In addition, studies examining surrogates of muscle mass [6, 7] as well as more accurate methods of estimation of muscle mass such as bioelectrical impedance spectroscopy (BIS) [8, 9] and dual-energy X-ray absorptiometry (DXA) [10] suggest that lower muscle mass is associated with higher mortality in this population. Given the increasing prevalence of obesity among ESRD patients over the last decade [11], recent studies have examined the presence of low muscle mass concurrent with high fat mass in the dialysis population (sarcopenic obesity) and found that sarcopenic obesity is associated with poor physical performance and lower extremity function [12].

The Kidney Disease Outcomes Quality Initiative (K/DOQI) guidelines recommend that all dialysis patients should be encouraged to increase their physical activity levels [13] based on evidence for the general population and for individuals at high risk of cardiovascular disease [14]. However, patients treated with dialysis are extremely sedentary, with activity levels falling below those reported by healthy sedentary individuals and by patients with other chronic diseases [15–17], and these extremely low levels of activity are associated with poor functional status and higher mortality [18, 19]. Higher physical activity is associated with higher muscle mass in dialysis [20, 21], and prior studies of exercise in dialysis patients have shown that vigorous exercise is associated with increase in muscle mass [19, 22, 23], as well as improvement in physical functioning [24–26]. However, the effect of a walking intervention on measures of body composition has not been studied.

We conducted a 6-month randomized controlled trial comparing 3 months of pedometers and weekly step goals plus a 3-month post-intervention follow up without counselling or pedometers with usual care among patients treated with dialysis [27]. We used bioelectrical impedance spectroscopy (BIS) to estimate body composition at 0, 3, and 6 months in order to ascertain whether the pedometer-based walking intervention would result in beneficial changes in body composition among patients on dialysis.

## Methods

Full details of recruitment and enrollment into the Pedometers and Exercise in Dialysis (PED) study have been previously described, as well as full details of testing of step counts [27].

### Inclusion and exclusion criteria

Patients were enrolled from three San Francisco dialysis clinics. Participants eligible for inclusion were ambulatory patients aged ≥18 years who were treated with in-center hemodialysis (HD) or any form of peritoneal dialysis (PD), and had telephone access. Patients using wheelchairs or scooters were excluded but those using a cane or other assistive device were eligible. Patients with pacemakers, intra-cardiac defibrillators, or metallic implants were excluded from body composition analysis. All patients provided informed consent to participate. The study was registered at ClinicalTrials.gov (NCT02623348) and approved by the UCSF Committee on Human Research (14–13,175).

### Baseline testing

Participants were asked basic demographic information such as race and ethnicity, and medical records were reviewed for information about comorbid conditions, laboratory results, medications, and dialysis prescription.

### Outcomes – body composition

Height was measured at baseline using a stadiometer. Weight was recorded to the nearest 0.1 kg from an average of two weights taken prior to body composition testing. Body-mass index (BMI) was derived from weight divided by height in meters-squared.

Patients treated with HD were assessed immediately before a mid-week dialysis session, and patients on PD were assessed at a usual clinic visit. For participants treated with PD, weight was recorded after first subtracting the weight from any in-dwelling dialysate. Body composition was estimated non-invasively using multi-frequency whole-body BIS performed with the SFB7 Body Composition Analyzer (ImpediMed), which scans 256 frequencies between 4 and 1000 kHz. Patients were asked to sit in a reclining chair and remain in that position for at least 5 min. Patients were asked to remove any jewelry, watches, or other metal objects and to position themselves such that no part of their body was making contact with or crossing over any other part. After cleaning the skin with an alcohol wipe, electrode pads were placed on their hands and feet and leads attached in the appropriate configuration for measuring resistance and reactance at various frequencies [28]. Total body muscle mass (TBMM) was estimated from intracellular fluid volume according to the following equation: TBMM (in kg) = 9.52 + 0.331 x BIS-derived intracellular volume (L) + 2.77 (male sex) + 0.180 x body weight (kg) – 0.113 x age (years) [29]. TBMM was then indexed to height in meters squared. Fat mass was estimated through the SFB7’s internal protocol by subtracting total body water (estimated using resistance extrapolated to infinite frequency) divided by 0.73 from body weight.

### Baseline step counts

Step counts were measured using pedometers (Accusplit AE120, Livermore, CA) [15, 30–32]. Patients were asked to wear the pedometer at their waist during waking hours for 1 week and to record a diary of daily steps. Study personnel obtained step counts by telephone or in-person visit.

### Randomization

Patients were randomly assigned to participate in a control group or our pedometer intervention program in a balanced 1:1 distribution, stratified by dialysis modality. We targeted enrollment of 48 HD patients and 12 PD patients, with sample size chosen to provide 80% power to detect an increase of 1000 steps or greater in the intervention group compared to the control group despite expected rates of dropout. Full details of randomization have been published previously [27].

### Intervention

The intervention consisted of providing pedometers and weekly counselling sessions in which a member of the study team called the participant each week at a scheduled time. Participants in the intervention group were asked to continue wearing their pedometers after baseline assessment and to record daily step counts for 3 months. During the weekly counselling session, participants relayed their diary of step counts from the prior week to research personnel who then provided goals for the upcoming week and advised about ways in which participants could increase daily walking. The initial counselling session was scheduled 1 week after baseline assessment and randomization.

Participants in the intervention group were recommended to increase their steps by 10% compared with the prior week. For patients who had hospitalizations or other events resulting in periods of reduced activity, we revised their goals such that they would increase by 10% increments starting at their new “baseline” daily step level.

Patients in the control group were asked to return the pedometers after relaying their record of steps from the initial week of data collection and were not contacted until the 3-month assessment. After the 3-month assessment, pedometers were returned to study personnel by both groups. We then measured step counts and body composition again after an additional 3 months.

### Statistical analysis

Patients’ baseline characteristics were summarized as median (25th, 75th percentile) for continuous variables or frequency and percentage for categorical variables. For step counts specifically, we calculated average daily steps over the week prior to each assessment for each participant and reported the mean of those average daily steps for each group. The primary outcome was between-group difference in change in measures of body composition at the end of the 6-month program. We used mixed effects linear regression analyses to assess changes at 6 months for TBMM, fat mass, and BMI. We adjusted for the stratification factor (dialysis modality), and sex, in each model. We also performed separate analyses using mixed effects linear regression analysis as above to assess changes from 0 to 3 months and from 3 to 6 months. In addition, we examined whether outcomes differed among HD and PD patients in a pre-specified subgroup analysis via an interaction test. We performed post-hoc analyses using linear regression to examine whether change in step counts from 0 to 3 months was associated with change in TBMM, fat mass, or BMI over the course of the 6-month program.

Two-sided *p*-values < 0.05 were considered statistically significant. Statistical analyses were performed using Stata, version 14 (StataCorp, College Station, TX).

## Results

### Baseline characteristics

**Fig. 1:**
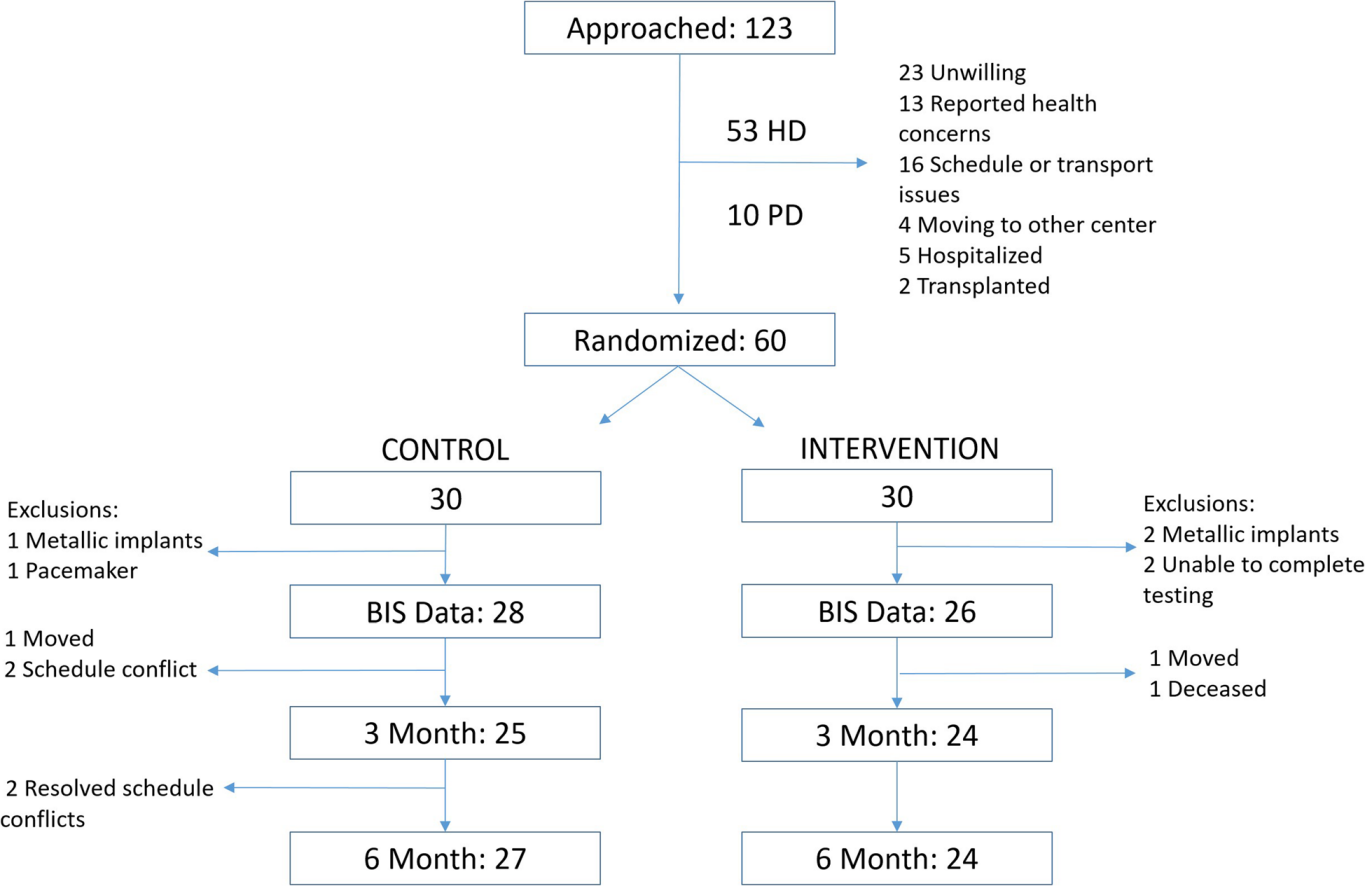
Recruitment, Randomization, and availability of BIS data

The first patient was enrolled in March 2016. We ceased enrollment after randomizing a total of 60 patients (48 HD and 12 PD) per our study protocol and the final assessment was performed in March 2018, concluding the study. We were able to obtain body composition measures on 44 HD and 10 PD patients (Fig. 1). The median age of patients with BIS data was 57.5 (53–66) and 76% were men. Thirty-nine percent of participants were black, 17% were white, and 40.7% had diabetes. Median dialysis vintage was 3.3 (1.2–5.8) years. The racial and ethnic distribution as well as comorbidities were similar between groups, but there were more men in the intervention group (Table 1). Therefore we adjusted for sex in our analysis. Baseline TBMM, fat mass, and BMI (Table 2) were similar in the intervention and control groups. Patients’ average step counts were 18,579 (8, 865–37,219) steps/week or 2631 (1209 – 5239) steps/day at baseline. Most patients’ median step counts classified them as sedentary (< 5000 steps/day).

**Table 1:** Patient characteristics at baseline for 44 hemodialysis patients and 10 peritoneal dialysis patients^a^

Characteristic	Control (***N*** = 28)	Intervention (***N*** = 26)
Age, years	56 (51, 66)	59.5 (53, 66)
Sex, % male	61	92
Race and Ethnicity, %		
White	18	15
Black	36	42
Asian	18	19
Hispanic	18	19
Other	14	15
Unknown/Unreported	11	8
BMI, kg/m^2^	31.6 (26.4, 34.6)	26.9 (25.3, 32.8)
Comorbidities, %		
HTN	93	92
DM	43	38
CAD	25	35
CHF	25	27
Stroke	14	8
Peripheral Vascular Disease	4	14
HIV	4	0
Arrhythmia	11	15
Dialysis Vintage, years	2.1 (0.96, 5.21)	3.8 (1.6, 7.2)
Hemoglobin, g/dL	10.9 (9.9, 11.6)	10.6 (9.8, 11.7)
Serum Albumin, g/dL	3.9 (3.7, 4.1)	3.9 (3.6, 4.0)
Std Kt/V	2.39 (2.11, 2.52)	2.30 (2.07, 2.44)
Education, %		
High School or Less	36	38
Vocational or Some College	32	27
College Degree	21	15
Professional or Graduate Degree	11	19
Currently smoking, %	36	15

^a^Values expressed in Median (25th, 75th percentile) or %

**Table 2:** Total body muscle mass (Adjusted for Height^2^), fat mass, and BMI in 44 hemodialysis patients and 10 peritoneal dialysis patients

	Baseline			Difference in change between groups from baseline to 3 months		Difference in change between groups from 3 to 6 months^a^		Difference in change between groups from baseline to 6 months^a^	
	Control (***N*** = 28) (25th, 75th percentile)	Intervention (***N*** = 26) (25th, 75th percentile)	***p***-value	Difference (95% CI)	***p*** -value**	Difference (95% CI)	***p*** -value**	Difference (95% CI)	***p*** -value**
**Total body muscle mass/height**^**2**^ **(kg/m**^**2**^**)**	10.3 (9.2, 11.4)	9.3 (8.3, 10.6)	0.65	0.3 (−0.1, 0.7)	0.14	0.4 (0.02, 0.9)	0.04	0.7 (0.3, 1.13)	< 0.01
**Fat mass (kg)**	25.1 (16.8, 34.3)	23.1 (16.4, 32.2)	0.90	−1.7 (−4.1, 0.8)	0.18	−2.6 (−5.3, − 0.1)	0.04	− 4.3 (− 7.1, − 1.5)	< 0.01
**BMI (kg/m**^**2**^**)**	31.6 (26.7, 34.6)	26.9 (25.3, 32.8)	0.45	−0.2 (− 1.0, 0.6)	0.58	− 0.8 (− 1.7, − 0.01)	0.05	−1.0 (− 1.8, − 0.2)	0.02

^a^Modeled through mixed-effects linear regression analysis

**p-value for between-group comparison of change, adjusted for sex and by stratification factor (modality)

### Change in outcomes at six months

Participants in the intervention group who had data available for this BIS analysis increased average daily steps by 2414 more steps (95% CI 1047 to 3782) from 0 to 3 months relative to controls, but during the 3 months after the active intervention phase decreased their step counts by 2429 (1016, 3841) compared to the control group. Therefore at 6 months there was no statistically significant difference in change in average daily step count between intervention and controls (49 steps, 95% CI − 1220 to 1317).

However, between 0 and 6 months, TBMM increased by 0.7 kg/m^2^ more in participants in the intervention than in the control group (95% CI 0.3 to 1.3). Conversely, fat mass and BMI decreased by 4.3 kg (95% CI 1.5 to 7.1) and 1.0 kg/m^2^ (95% CI 0.2 to 1.8) more than in the control group, respectively (Table 2). The effect of the intervention did not differ by modality (HD versus PD) for any outcome (*P* for interaction > 0.9).

When examining individual time points, at 3 months TBMM for participants in the intervention had increased by 0.30 kg/m^2^ more than in the control group (− 0.1, 0.7) and fat mass and BMI had decreased by − 1.7 kg (− 4.1, 0.8) and − 0.2 kg/m^2^ (− 1.0, 0.6) more than in the control group, respectively. However, no difference in change in body composition between 0 and 3 months achieved statistical significance. Between 3 and 6 months, participants in the intervention increased TBMM by 0.4 kg/m^2^ (95% CI 0.02 to 0.9), decreased fat mass by − 2.6 kg (95% CI − 5.3 to − 0.1), and decreased BMI by − 0.8 kg/m^2^ (95% CI − 1.7 to − 0.01) as compared to controls.

### Post-hoc analysis of association of change in step counts with change in body composition measures

When examining the entire study cohort, each increase of 1000 steps per day from 0 to 3 months was associated with a decline in fat mass of − 0.3 kg (95% CI − 0.5 to − 0.05) over the 6-month period of the study (Table 3). There was no statistically significant association between change in step count from 0 to 3 months and change in TBMM or change in BMI.

**Table 3:** Association of change in step count from baseline to 3 months with change in total body muscle mass, fat mass, and BMI through linear regression analysis

Unadjusted				Adjusted^a^		
	Change in total body muscle mass/height^**2**^ (kg/m^**2**^)	Change in fat mass (kg)	Change in BMI (kg/m^**2**^)	Change in total body muscle mass/height^**2**^ (kg/m^**2**^)	Change in fat mass (kg)	Change in BMI (kg/m^**2**^)
**Change in average daily Step Count (per 1000 steps)**	0.01 (− 0.02, 0.05)	−0.3 (− 0.6, − 0.1)	−0.04 (− 0.1, 0.03)	0.02 (− 0.02, 0.05)	−0.3 (− 0.5, − 0.05)	−0.02 (− 0.1, 0.06)
***p*-value**	0.44	< 0.01	0.28	0.39	0.02	0.57

^a^Adjusted for age, sex, and modality

### Safety monitoring and adverse events

Of patients included in this analysis, six participants (23.1%) reported symptoms related to the intervention. Symptoms included shortness of breath (7.6%), soreness (7.6%), lower extremity pain (3.8%), cramping (11.5%), and fatigue during or after walking (7.6%). Two patients reported chest pain with walking and were advised to walk only as much as they usually would until they were able to consult their cardiologists. One patient (in the intervention group) died during the study, but the death was determined not to be related to the intervention. There were no hospitalizations related to the intervention, nor was there any significant difference between groups in hospitalizations within the last 6 months at time of study completion.

## Discussion

**Fig. 2:**
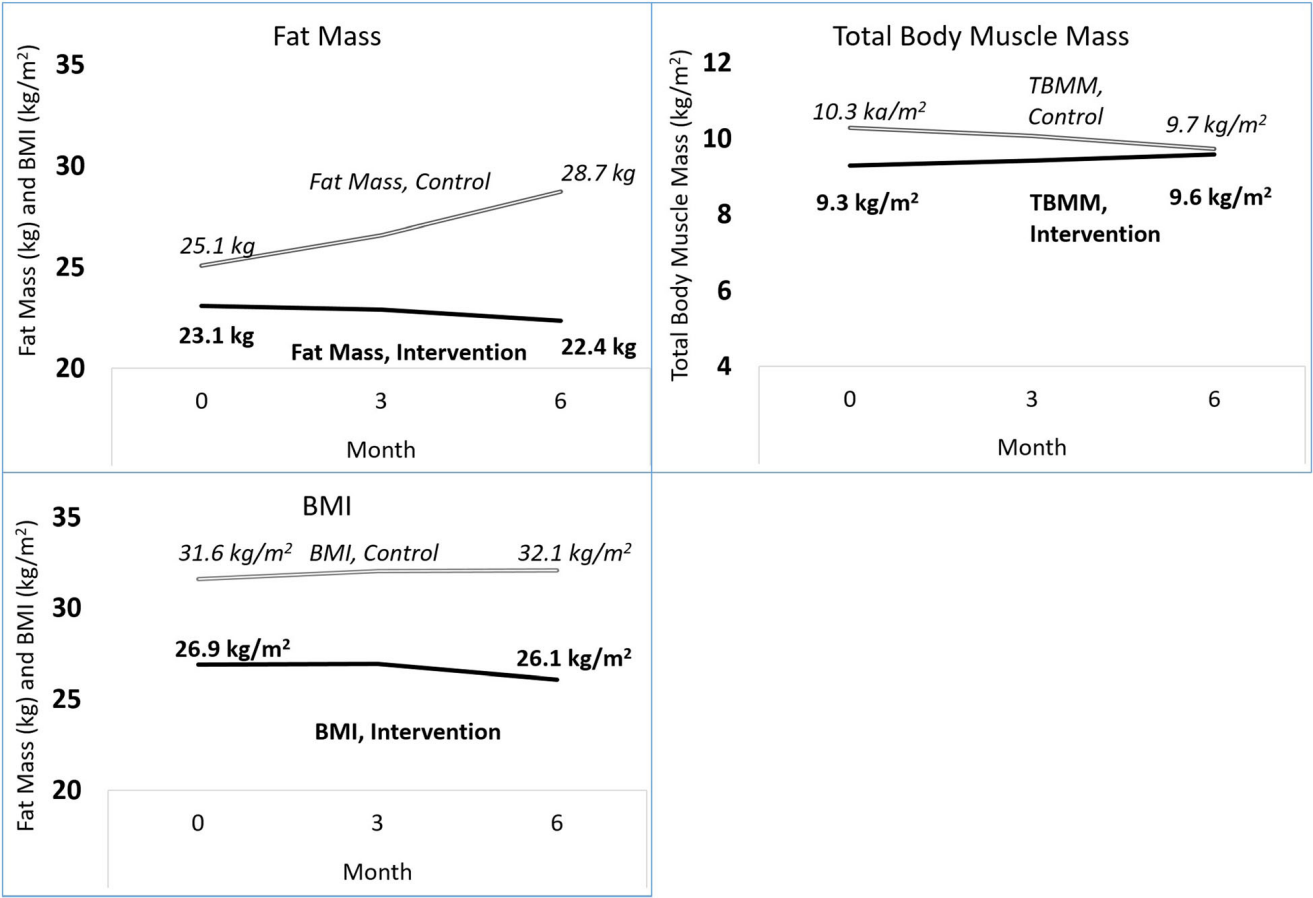
Body Composition in 44 HD and 10 PD patients at 0, 3, and 6 months

Participants in this study were sedentary when compared to other studies of patients treated with dialysis [20, 21] as well as studies measuring steps in other chronic disease populations [15]. Participants in the interventional group had a modest and statistically significant increase in step counts from baseline to 3 months compared with controls but at 6 months had regressed back to baseline levels of walking. Despite this regression, participants in the intervention group had a significantly greater increase in TBMM, decrease in fat mass, and decrease in BMI relative to controls from 0 to 6 months (Fig. 2). There was a dose-response relationship between steps added from 0 to 3 months and loss in fat mass over the 6-month period of the study but no similar relationship with TBMM or with BMI. Weight loss appeared primarily driven by changes in fat mass with relative preservation of muscle mass, and the difference between groups was a combination of improvement in the interventional group and negative changes in the control group.

Measurements of body mass tend to decline over time in prevalent ESRD cohorts due in large part to the effects of malnutrition and inflammation [33, 34]. Having higher muscle or lean body mass has been associated consistently with survival benefits in patients with kidney disease and particularly in those receiving dialysis [8, 9, 35]. Protein-energy wasting is itself an independent predictor of mortality and is associated with not only a loss of lean body mass but also in increased fat mass. However, having a higher fat mass appears to be associated with lower mortality in patients with kidney disease [35, 36]. Furthermore, loss of fat mass has been associated with higher mortality in the dialysis population [37, 38], although it is not clear whether fat *gain* is protective. More recent studies of discordant changes in muscle and fat mass show that this “obesity paradox” may be explained more by changes in muscle mass than fat mass [6], and having high BMI with low muscle mass is associated with higher mortality than normal BMI with low muscle mass or high BMI with high muscle mass [7]. It is possible that gain of muscle mass with overall weight loss may confer additional survival benefit over weight gain alone [36].

Our study shows that a walking intervention not only prevented decline in muscle mass but also resulted in a slight increase. Prior studies of moderate or vigorous resistance exercise training in dialysis patients have shown significant improvements in muscle mass [19, 22, 23], and it may be that more vigorous exercise training results in greater increase in muscle mass [39]. However, only a fraction of dialysis patients are able to participate in such interventions [40]. A walking program may be more manageable, and if such an intervention were sustained for a longer period of time, further improvement in muscle mass may be possible. However, the fact that participants in the intervention regressed in their step counts from 3 to 6 months has important implications on the importance of feedback to sustaining walking. If such an intervention were scaled up, it may be necessary to implement more automated or technology-driven methods to allow for longer interventions without requiring weekly personal contact. Our study also demonstrates a decrease in fat mass in patients in the intervention group as compared to an increase in fat mass in controls.

Although the between-group differences in change in BIS measures at 6 months appears to be driven at least as much by decrease in muscle mass and increase in fat mass in the controls, it is still surprising that total body muscle mass continued to increase and fat mass continued to decrease in the intervention group despite patients having regressed back to baseline levels of walking at 6 months. In fact, the between-group differences in change in body composition were larger from 3 to 6 months than from 0 to 3 months. It may be that the intervention group continued to have higher step counts for some portion of the follow-up period. However, as we did not measure weekly step counts in between the 3- and 6-month assessments, we cannot ascertain whether step counts immediately declined after study personnel ceased providing feedback or whether participants were able to maintain their steps for a greater length of time. It is also possible that the period of sustained exercise during the active portion of the intervention resulted in a “legacy effect” sustained even after patients’ step counts decreased back towards baseline values. Though this type of effect has been described in other studies of exercise, they have been more vigorous in intensity and also of a longer duration [41]. However, the fact that the number of steps gained in the intervention period was associated significantly with change in fat mass supports that the initial ramp-up of activity played some role in the differences in BIS measures at 6 months. Inactivity is associated with muscle atrophy [42], and the modest increase in steps during the intervention period may have prevented wasting. Kidney disease is also associated with a chronic inflammatory state and significant protein-energy wasting [43–45], and physical activity and exercise have been found to have anti-inflammatory effects [46].

It should also be noted that step counts are a light-intensity activity and do not capture the full spectrum of physical activity. Therefore another possibility is that though walking regressed from 3 to 6 months, patients engaged in isotemporal substitution with other forms of activity, and so some portion of the continued increase in muscle mass and decrease in fat mass in the intervention group was from reduction in overall sedentary time rather than walking [47]. However, walking is the most common form of physical activity for most dialysis patients [48], and it is not evident that patients would switch their focus to other activities so readily. It is possible that activity of even low to moderate intensity could result in reduced protein-energy wasting for a period of time even after cessation of activity. Indeed, sedentary behavior and protein-energy wasting have independent but synergistic effects on muscle [49].

This study has several limitations. We acknowledge that more detailed information on the trajectory of step counts between 3 and 6 months as well as data on other types of activity that patients engaged in would be valuable in answering questions about the appropriate dose and duration of activity that we should recommend to patients. Due to timing and patient convenience, we performed measurements of BIS immediately before hemodialysis when patients were not at their “dry” weight. However, we did attempt to minimize variability by performing all testing for hemodialyiss patients on a mid-week dialysis day and at the same time prior to their scheduled dialysis sessions at all time points. In addition, the equation used to estimate total body muscle mass is derived from pre-dialysis measurements of BIS [29]. Furthermore, our outcome was the difference in change between groups. Study participants were selected from dialysis facilities in the United States, who may be more sedentary compared to patients treated with dialysis elsewhere. However, it is not clear that the baseline level of activity would affect the observed association of increased walking with beneficial changes in body composition. Finally, despite randomization, there were more men in the interventional group. However, performing this analysis as a difference in change between groups mitigates the effect of any baseline imbalances.

## Conclusions

Patients assigned to a pedometer-based intervention lost weight compared with patients who did not engage in the intervention, and this weight loss was driven primarily by reduction in fat mass with relative preservation of muscle mass. Achieved changes in step counts were correlated with changes in fat mass. Walking interventions using simple and inexpensive devices such as pedometers coupled with feedback on activity goals have the potential to not only increase activity but also to result in beneficial changes in body composition. Further study is needed to determine whether such changes can be maintained over a longer duration or whether they are of sufficient magnitude to result in improvement in physical function or survival.

## Data Availability

Individual participant data that underlie the results reported in this article will be shared upon reasonable request, after deidentification (text, tables, figures and appendices), beginning 9 months and ending 36 months following article publication, with investigators whose proposed use of the data has been approved by an independent review committee (“learned intermediary”) identified for the purpose of individual participant data meta-analysis. Proposals may be submitted up to 36 months following article publication and should be directed to Dr. Anoop Sheshadri (Anoop.Sheshadri@ucsf.edu).

## Abbreviations

BIS: Bioelectrical impedance spectroscopy
DXA: Dual-energy X-ray absorptiometry
BMI: Body-mass index
ESRD: End-stage renal disease
HD: Hemodialysis
K/DOQI: Kidney Disease Outcomes Quality Initiative
PD: Peritoneal dialysis
TBMM: Total body muscle mass
